# Pollen Morphology in *Sorbus* L. (Rosaceae) and Its Taxonomic Implications

**DOI:** 10.3390/plants12183318

**Published:** 2023-09-20

**Authors:** Meng Li, Chang-Fen Tian, Muhammad Idrees, Mitra Pathak, Xian-Hua Xiong, Xin-Fen Gao, Xian-Rong Wang

**Affiliations:** 1 Co-Innovation Center for Sustainable Forestry in Southern China, College of Biology and the Environment, Nanjing Forestry University, Nanjing 210037, China; limeng@njfu.edu.cn (M.L.);; 2 College of Life Science, Neijiang Normal University, Neijiang 641000, China; idrees@njtc.edu.cn; 3 Plant Research Center, Salyan, Department of Plant Resources, Ministry of Forests and Environment, Kathmandu 44710, Nepal; scientistdrmitra@gmail.com; 4 College of Life Science and Biotechnology, Mianyang Teachers’ College, Mianyang 621000, China; xianhua007@126.com; 5 Chengdu Institute of Biology, Chinese Academy of Sciences, Chengdu 610041, China; xfgao@cib.ac.cn

**Keywords:** Maleae, palynology, scanning electron microscopy, cluster analysis

## Abstract

The genus *Sorbus* L. in the Rosaceae family is taxonomically challenging due to its morphological variation, polyploidy, and interspecific hybridization. In this study, we used scanning electron microscopy (SEM) to observe the pollen morphology of eighty species, representing six subgenera, in order to assess the differences within the genus *Sorbus* and its pollen characteristics. We conducted a cluster analysis on three qualitative and four quantitative characteristics. The results demonstrated that the pollen grains of the studied *Sorbus* species are isopolar and tricolporate. We identified five types of pollen shapes: suboblate, spheroidal, subprolate, prolate, and perprolate. The pollen ornamentation of the investigated species could be classified into five types: striate-perforate, striate, cerebroid-perforate, cerebroid, and foveolate. Interestingly, within the same subgenera, different species exhibited multiple types of characters. The cluster analysis indicated that all 80 species could be divided into six groups, with group B consisting exclusively of species from the subgenus *Sorbus*. Although pollen micro-morphologies alone do not provide sufficient evidence to establish the taxonomic relationships of the subgenera within *Sorbus*, they do offer valuable information for species-level taxonomic treatment.

## 1. Introduction

The genus *Sorbus* L., which belongs to the family Rosaceae, is of significant economic and ornamental importance. It comprises 258 species and is distributed across Europe, Africa, Asia, and America [1,2]. The East Asia region is considered the center of distribution for *Sorbus*. Due to factors such as intraspecific variation, polyploidy, and interspecific hybridization, species circumscription within this genus is highly challenging [1,3,4]. Initially, Linnaeus only included two compound-leaved species when he first published about this genus [5]. However, with the advancement in systematic botany, further studies have identified additional species. Some taxa of *Sorbus* have been merged into other genera of Maleae, such as *Pyrus* L. and *Crataegus* L., and considered as subgenera or sections [2,6]. There has been a debate among taxonomists regarding whether *Sorbus* solely comprises compound-leaved species [7,8,9,10,11] or includes both single-leaved and compound-leaved taxa [3,12,13,14,15,16].

The genus *Sorbus* has been classified into six subgenera (*Sorbus*, *Cormus* Spach, *Aria* (Pers.) Host, *Micromeles* Decne., *Torminaria* M. Roem., and *Chamaemespilus* Medik.) based on morphological characteristics [1]. However, other studies suggest that *Sorbus* species can be organized into three sections [16], eleven sections [17], or five groups [6,18]. Recent studies utilizing DNA sequence data in phylogenetic analyses have shown that *Sorbus* sensu lato is polyphyletic and *Sorbus* sensu stricto is monophyletic [19,20,21,22,23]. Nevertheless, the infraspecific relationship among the other five subgenera remains unclear. For instance, subgenus *Micromeles* has been considered both a subgenus within *Sorbus* [1,16] and a distinct genus [6,24]. Recent research has indicated that hybridization may have occurred between the ancestral lineages of *Sorbus* and *Aria* prior to the early Miocene, suggesting that they were the most likely parents of *Micromeles* [20]. As a result, five hybridogenous genera of *Sorbus s.l.* have been established based on phylogenetic studies, and the name *Sorbomeles* has been proposed for hybrids of *Sorbus* and *Micromeles* [2]. Rushforth has accepted the six genera published by previous taxonomists, as well as the additional five genera described by Sennikov and Kurtto [2] and has proposed an additional five new genera of Asiatic whitebeams [25]. Consequently, further study is needed to provide reliable taxonomic evidence for the classification of *Sorbus*.

Pollen morphology is a conserved feature that remains unaffected by changes in environmental conditions, making it a valuable tool for taxonomic classification and identification of plants. Recent advancements in microscopic technology have enabled the observation of pollen surface sculptures, facilitating the resolution of taxonomic problems. Numerous studies in palynology have demonstrated that pollen observation provides effective evidence for the classification and identification of plants at both the generic and specific levels [26,27,28,29,30,31,32,33,34].

Although some research papers have examined and published the pollen morphology of certain *Sorbus* species [35,36,37,38,39,40], the overall body of work in this area remains limited, often focusing on minority species or specific taxa. Currently, there is a lack of comprehensive documentation regarding the pollen morphology of the *Sorbus* genus in both China and the rest of the world. Therefore, our study aimed to address these gaps by sampling eighty species across all six subgenera of *Sorbus*. The specific objectives were to: (1) observe and describe the pollen morphology using scanning electron microscopy (SEM), (2) analyze all collected data and discuss the taxonomic significance of the pollen morphology within *Sorbus* subgenera and among different species, and (3) explore the correlations between the observed pollen morphology and previous taxonomic research conducted on *Sorbus*.

## 2. Results

### 2.1. Pollen Size and Shape

**Figure 1:**
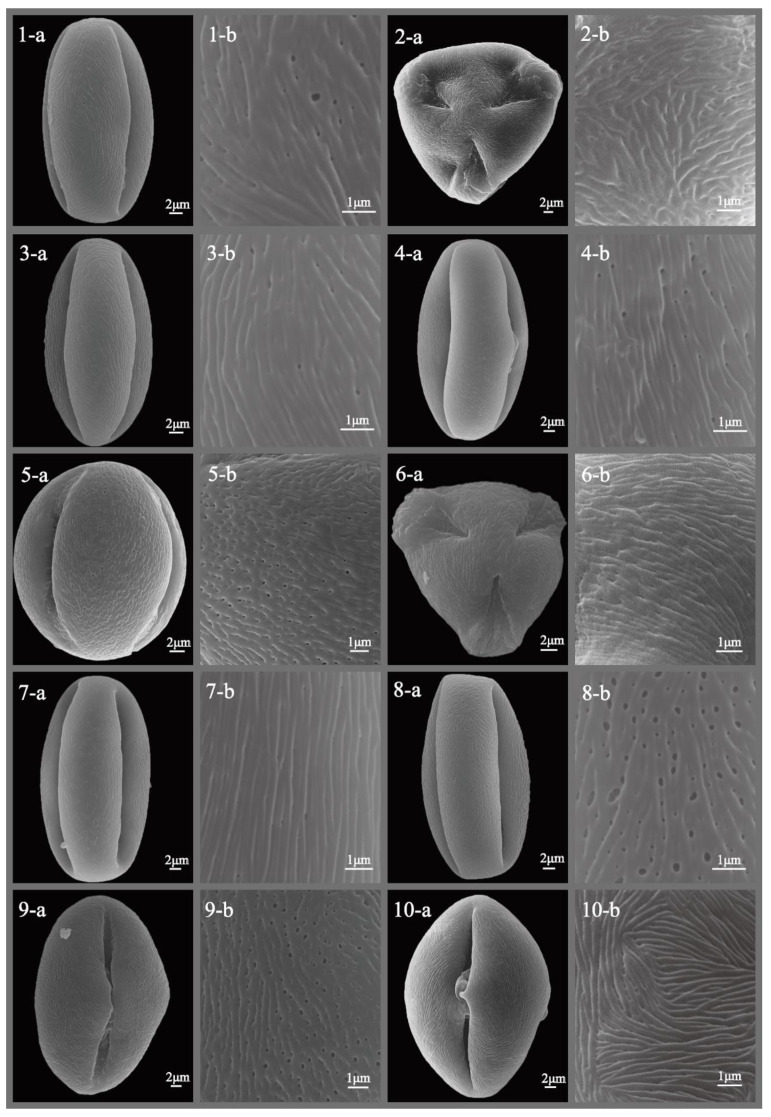
SEM micrographs of pollen grains of studied *Sorbus* taxa. (**1-a**–**10-a**) Pollen grains in polar view. (**1-b**–**10-b**) Pollen ornamentation. 1, *S. albovii*; 2, *S. alnifolia*; 3, *S. americana*; 4, *S. amurensis*; 5, *S. aira*; 6, *S. aucuparia*; 7, *S. boissieri*; 8, *S. buschiana*; 9, *S. californica*; 10, *S. caloneura*.

**Figure 2:**
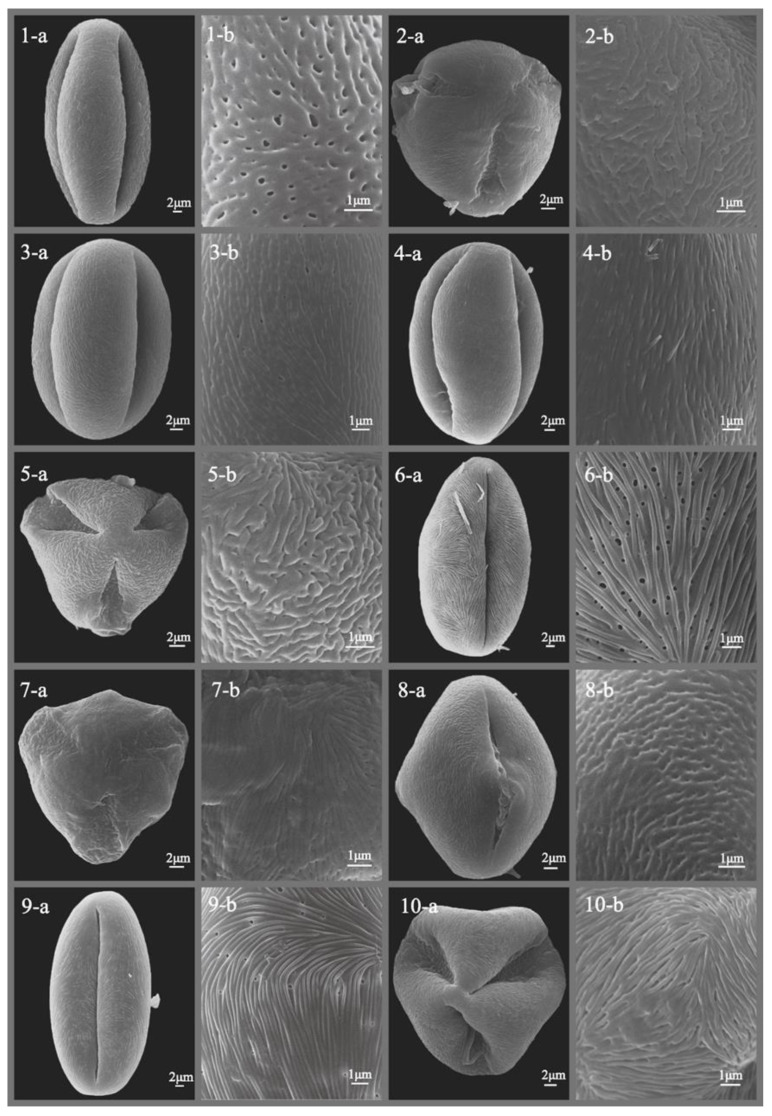
SEM micrographs of pollen grains of studied *Sorbus* taxa. (**1-a**–**10-a**) Pollen grains in polar view. (**1-b**–**10-b**) Pollen ornamentation. 1, *S. caucasica*; 2, *S. chamaemespilus*; 3, *S. commixta*; 4, *S. corymbifera*; 5, *S. decora*; 6, *S. devoniensis*; 7, *S. discolor*; 8, *S. domestica*; 9, *S. dunnii*; 10, *S. esserteauiana*.

**Figure 3:**
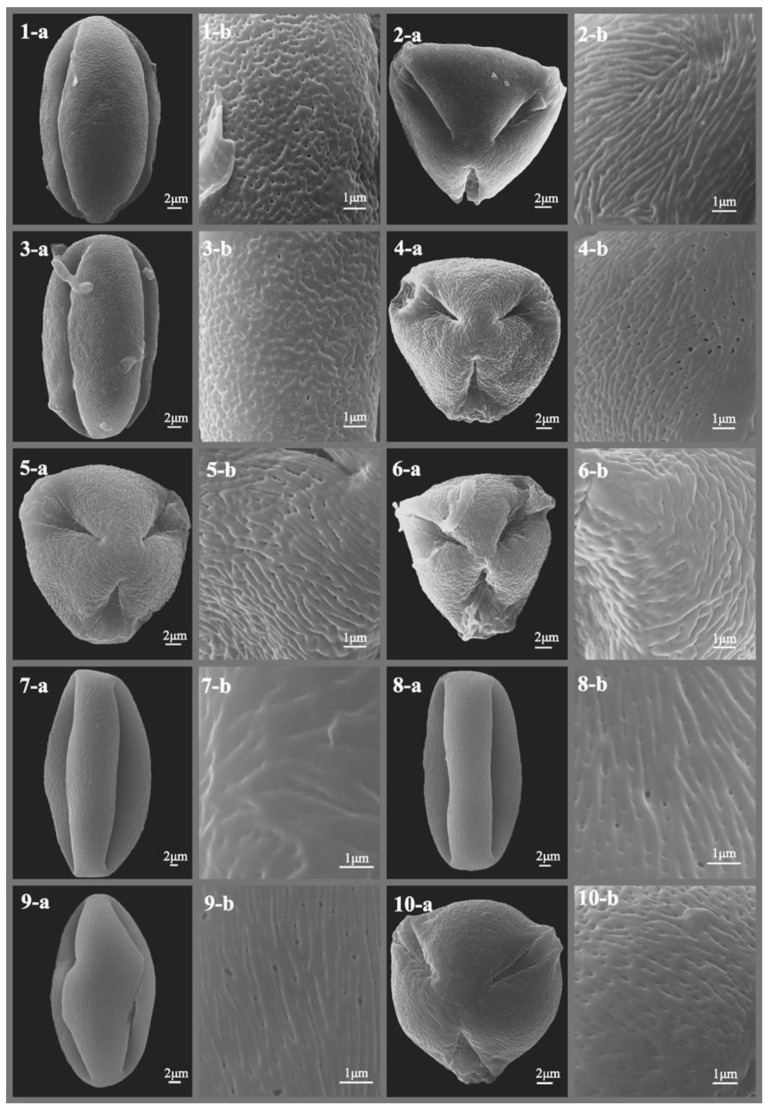
SEM micrographs of pollen grains of studied *Sorbus* taxa. (**1-a**–**10-a**) Pollen grains in polar view. (**1-b**–**10-b**) Pollen ornamentation. 1, *S. folgneri*; 2, *S. foliolosa*; 3, *S. gracilis*; 4, *S. graeca*; 5, *S. harrowiana*; 6, *S. helenae*; 7, *S. hemsleyi*; 8, *S. hupehensis*; 9, *S. hybrida*; 10, *S. insignis*.

**Figure 4:**
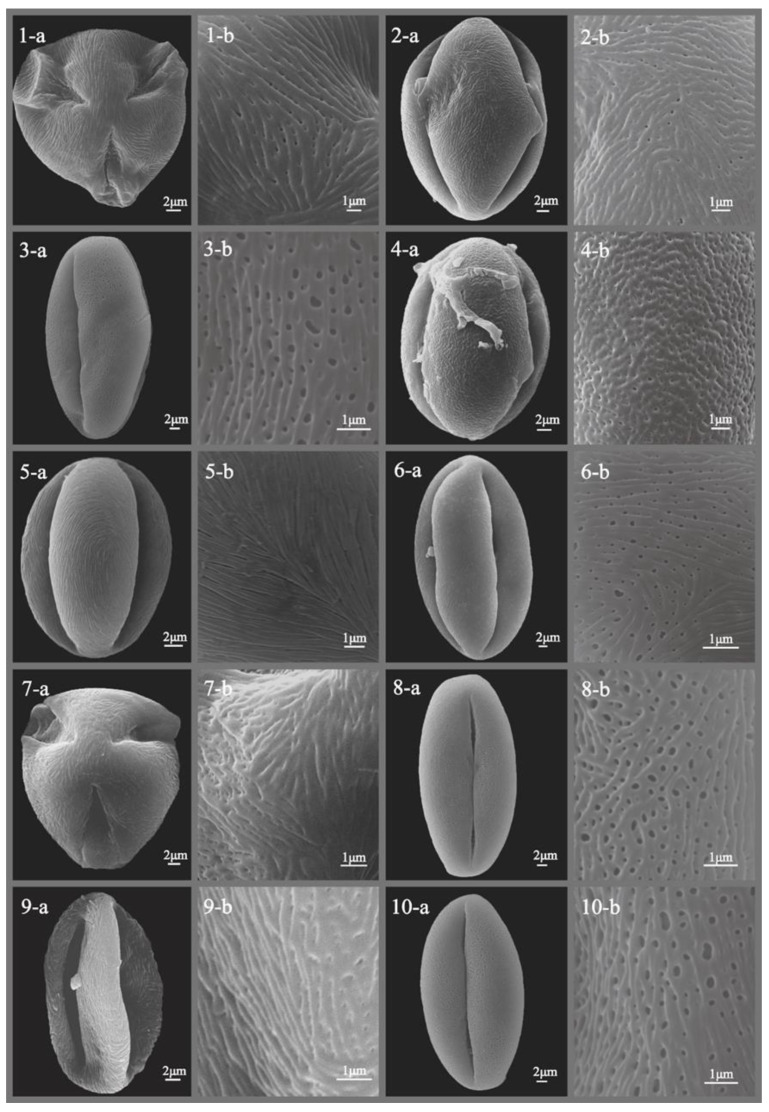
SEM micrographs of pollen grains of studied *Sorbus* taxa. (**1-a**–**10-a**) Pollen grains in polar view. (**1-b**–**10-b**) Pollen ornamentation. 1, *S. intermedia*; 2, *S. japonica*; 3, *S. keissleri*; 4, *S. koehneana*; 5, *S. kurzii*; 6, *S. latifolia*; 7, *S. matsumurana*; 8, *S. monbeigii*; 9, *S. mougeotii*; 10, *S. persica*.

**Figure 5:**
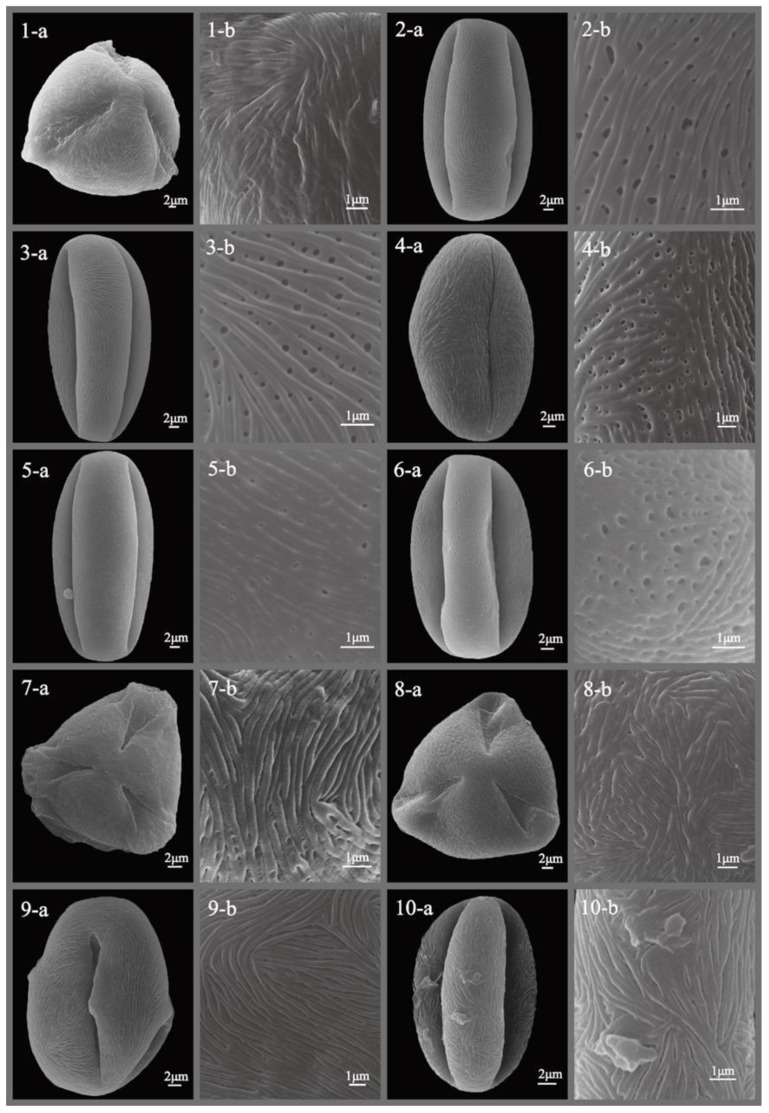
SEM micrographs of pollen grains of studied *Sorbus* taxa. (**1-a**–**10-a**) Pollen grains in polar view. (**1-b**–**10-b**) Pollen ornamentation. 1, *S. pohuashanensis*; 2, *S. prattii*; 3, *S. rehderiana*; 4, *S. rufo-ferruginea*; 5, *S. sambucifolia*; 6, *S. sargentiana*; 7, *S. scopulina*; 8, *S. sibirica*; 9, *S. sitchensis*; 10, *S. tauricola*.

**Figure 6:**
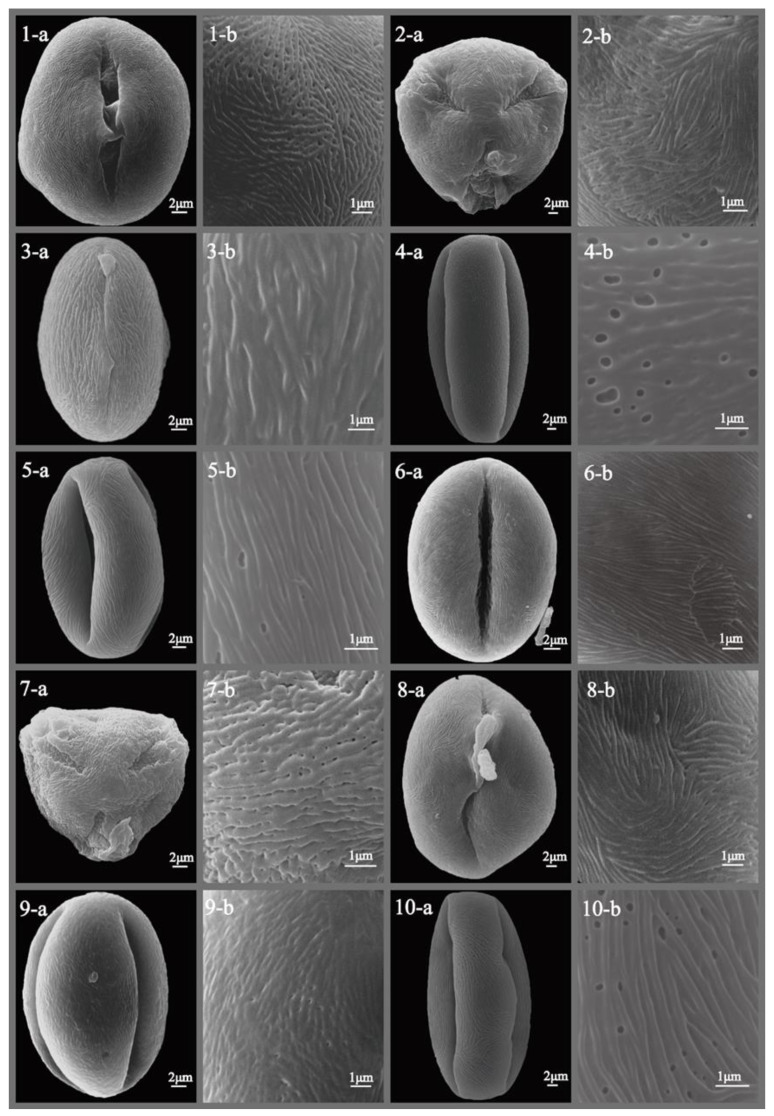
SEM micrographs of pollen grains of studied *Sorbus* taxa. (**1-a**–**10-a**) Pollen grains in polar view. (**1-b**–**10-b**) Pollen ornamentation. 1, *S. thibetica*; 2, *S. thomsonii*; 3, *S. tianschanica*; 4, *S. torminalis*; 5, *S. tsinlingensis*; 6, *S. umbellate*; 7, *S. ursine*; 8, *S. verrucosa*; 9, *S. vilmorinii*; 10, *S. yuana*.

The pollen morphology of all studied *Sorbus* species was thoroughly described and illustrated with SEM photographs (Figure 1, Figure 2, Figure 3, Figure 4, Figure 5 and Figure 6), revealing predominantly small- to medium-sized pollen grains. Among the six subgenera examined, medium-sized pollen grains were observed in all, while small-sized pollen grains were specifically found in subgenera *Sorbus*, *Micromeles*, and *Aria*.

On average, the length of the polar axis (P) ranged from 16.64 μm to 50.70 μm, with the entire range extending from 15.59 μm to 54.08 μm. Notably, the longest polar axis length was observed in *S. devoniensis* of subgenus *Aria*, while the shortest was observed in *S. sargentiana* of subgenus *Sorbus*. Regarding the equatorial axis (E), the mean value varied between 11.58 μm and 32.38 μm, with the entire range spanning from 10.53 μm to 35.06 μm. The species with the longest equatorial axis length was *S. tauricola* within subgenus *Aria*, whereas the shortest was found in *S. albopilosa* of subgenus *Sorbus*.

The length of colpi ranged from 12.92 μm (*S. graeca*) to 43.60 μm (*S. devoniensis*). The C/P ratio demonstrated relative stability, with the majority of species (82.5%) falling within the range of 0.71 to 0.90 μm.

All pollen grains were isopolar and the of P/E ratio varied from 0.83 to 2.09. According to the classification rules, more than half of the pollen grains showed prolate shapes (51.25%). The others appeared in four shapes: suboblate (8.75%), spheroidal (16.25%), subprolate (13.75%), and perprolate (10.00%). Except for subgenera *Cormus*, *Torminaria*, and *Chamaemespilus*, the other subgenera (*Micromeles*, *Sorbus*, and *Aria*) revealed three or five different pollen types.

### 2.2. Pollen Ornamentation

**Figure 7:**
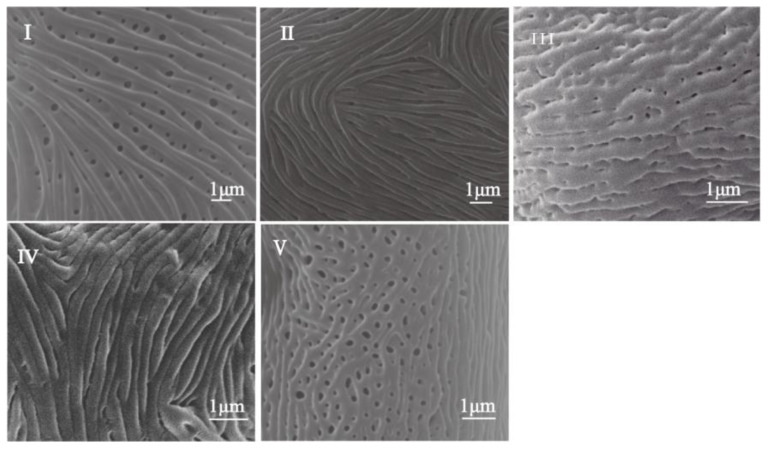
Scanning Electron Microphotographs of pollen ornamentation types. (Type **I**), striate-perforate; (Type **II**), striate; (Type **III**), cerebroid-perforate; (Type **IV**), cerebroid; (Type **V**), foveolate.

The variations of all characters selected in this study across subgenera and detailed pollen morphological feature data of the investigated taxa are shown (Table 1 and Supplementary Table S1). According to the diverse morphological characteristics of the examined specimens, the pollen ornamentation was classified into five types (Figure 7): I, striate-perforate, II, striate, III, cerebroid-perforate, IV, cerebroid, and V, foveolate.

Type I: Striate-perforate pollen grains were characterized by irregularly distributed parallel stripes and holes of different sizes on the surface. This type was observed in 29 species belonging to subgenera *Aria*, *Sorbus*, and *Micromeles*, with a hole density of less than 2.54 per μm.

Type II: Striate pollen grains were similar to type I in terms of parallel stripes on the surface, but they lacked perforations. The stripes and grooves ran parallel to the polar axis and often formed fingerprint-like twists. Twenty-five taxa from three subgenera were assigned to type II, including species like *S. persica* from subgenus *Aria*, *S. foliolosa*, and *S. multijuga* from subgenus *Sorbus*, and *S. thomsonii* from subgenus *Micromeles*.

Type III: Pollen grains of this type exhibited an exine ornamentation that bent into a brain shape with small holes of less than 1 μm in diameter uniformly distributed between lines and grooves on the pollen surface. Twelve species from four subgenera were found to have this type of ornamentation.

Type IV: This type was characterized by regular brain-like stripes on the pollen surface without any holes. Seven species from subgenus *Sorbus*, as well as *S. japonica* from subgenus *Micromeles*, were assigned to this type.

Type V: Foveolate pollen grains were distinct, with evenly distributed holes across the entire surface and few prominent lines. Seven species, including *S. torminalis* from subgenus *Torminaria*, *S. domestica* from subgenus *Cormus*, and five species from subgenus *Sorbus* such as *S. sitchensis*, exhibited this type of ornamentation.

### 2.3. Cluster Analysis of Sorbus Based on Measured Data

**Figure 8:**
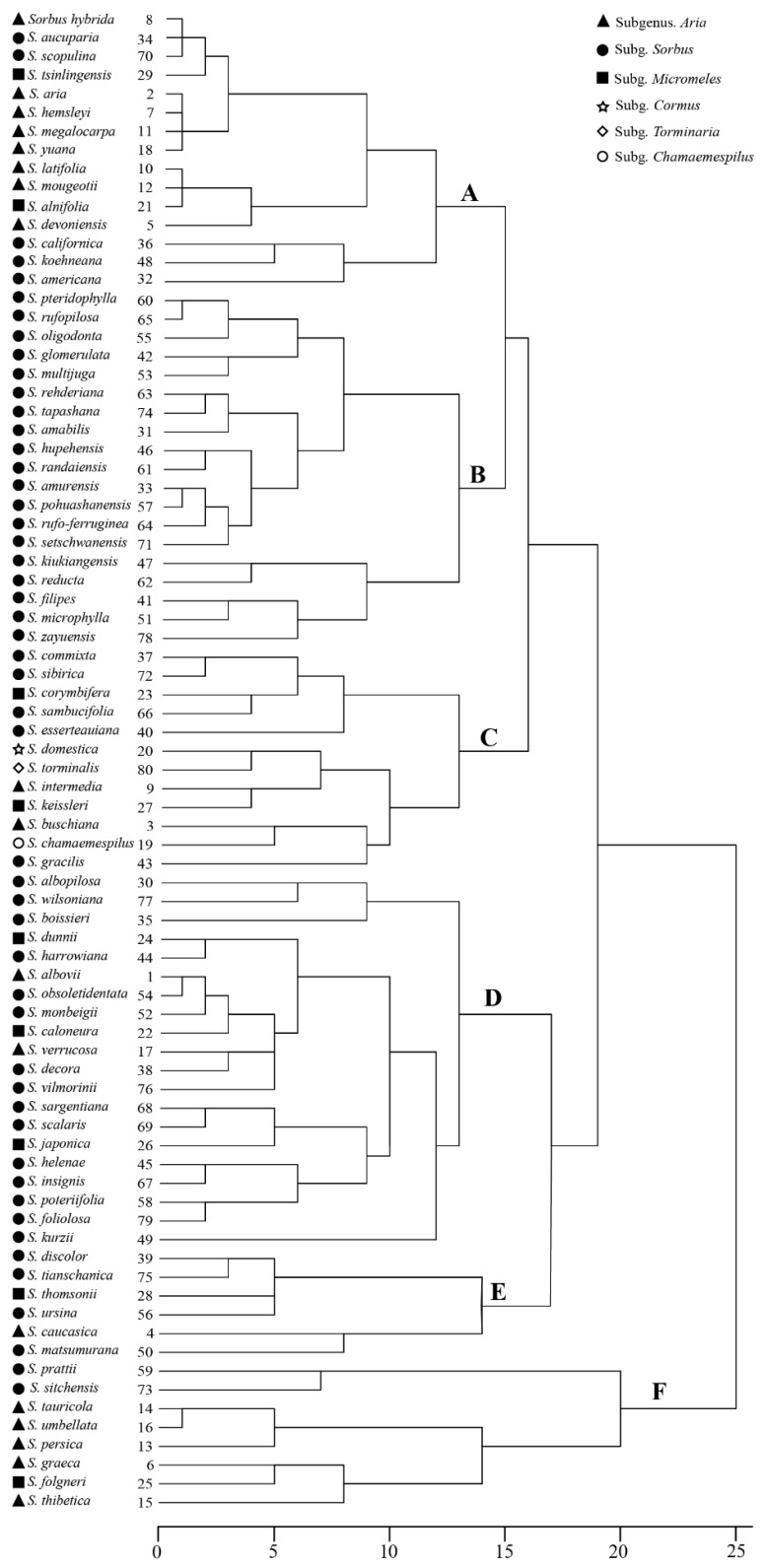
The phenogram of cluster analysis is based on 7 characters.

Cluster analysis was conducted using three qualitative and four quantitative characters (Figure 8), resulting in the classification of the examined *Sorbus* species into six distinct groups (A, B, C, D, E, F). In the cluster tree, all tested species showed intersections with one another, and taxa from the same subgenus did not form separate branches. With the exception of Group B, the other groups consisted of taxa from multiple subgenera.

Group A consisted of fifteen species from three subgenera: *Sorbus*, *Micromeles*, and *Aria*, and significant differences were observed among them. The majority (86.67%) of species in Group A exhibited striate-perforate ornamentation and had prolate or perprolate pollen shapes.

Group B was the only group that exclusively consisted of 19 species from subgenus *Sorbus*. It was characterized by striate or striate-perforate ornamentation with prolate-shaped pollen grains. The quantitative traits of pollen from these 19 taxa were greater than those of other subgenus *Sorbus* taxa (P: 23.54–35.38 μm, E: 13.77–27.59 μm, C: 21.27–30.93 μm, C/P: 0.81–0.98 μm).

Group C comprised twelve species from all six subgenera of *Sorbus*, and was characterized by cerebroid-perforate or foveolate ornamentation. The pollen grains in this group were prolate with longer polar axis lengths (27.07–40.86 μm) and high hole density.

Group D consisted of fifteen taxa from subgenus *Sorbus* and five taxa from subgenera *Aria* and *Micromeles*, and was characterized by imperforate pollen grains.

Group E included species from subgenera *Sorbus*, *Micromeles*, and *Aria*. In comparison to other branches, this group exhibited shorter polar axis lengths.

Group F comprised five taxa from subgenera *Aria*, *Micromeles*, and *Sorbus*, with pollen grains exhibiting suboblate or spheroidal shapes.

## 3. Discussion

In this study, our focus was on the pollen micro-morphology of the genus *Sorbus*. The observed variations in pollen ornamentation have provided valuable information. Overall, there is a high degree of similarity in pollen ornamentation at the genus level. However, certain differences have been identified at the subgenus or species level when compared to previous studies.

### 3.1. Pollen Size and Shape

Regarding pollen size and shape, all the investigated species had small- or medium-sized pollen grains with three apertures, consistent with earlier research. The shape of the pollen grains displayed significant variation, with the majority being spheroidal, subprolate, or prolate, while a few species exhibited suboblate or perprolate shapes.

The mean value of the polar axis length ranged from 16.64 μm to 50.70 μm, and the equatorial axis length ranged from 11.58 μm to 32.38 μm. Previous studies on the pollen morphology of *Sorbus* have reported mean polar axis lengths ranging from 16.47 μm to 45.0 μm, and equatorial axis lengths varying from 9.89 μm to 34.2 μm [35,36,37,39,40].

Bednorz et al. [36] reported that the polar axis length of *S. aria*, *S. torminalis*, *S. intermedia*, and *S. chamaemespilus* was typically over 28 μm with some slight overlap. However, in our study, the polar axis length of these species was consistently over 32 μm with relatively stable ranges of variability. In comparison, Yang’s observations of pollen grains showed larger polar and equatorial axis lengths, with D values of up to 12 μm [35]. It is evident that there are some discrepancies between our results and previous studies, particularly in terms of the significant variation in polar and equatorial axis lengths within the same *Sorbus* species. Previous articles have primarily focused on a limited number of taxa within *Sorbus*, which may explain the major differences found between our findings and theirs. These findings suggest that pollen size is not a stable characteristic, and that there exists individual variation in pollen grains among *Sorbus* species. The availability of materials and the selection of species to study are likely critical factors in palynological research on *Sorbus*.

### 3.2. Pollen Ornamentation

Ornamentation is a significant distinguishing characteristic in rosaceous pollen, and researchers have regarded pollen ornamentation as the foundation for *Sorbus* classification [30,35,39,40,41]. In this study, striate sculpture was observed in 54 species, with the highest occurrence in subgenera *Sorbus* and *Aria*. Cerebroid sculpture was found in nineteen species belonging to four subgenera (*Sorbus*, *Micromeles*, *Aria*, and *Chamaemespilus*), and faveolated pollen was found in seven species belonging to subgenera *Sorbus*, *Torminaria*, and *Cormus*. Previous studies by other scholars have likewise identified sculpture, cerebroid, and occasional faveolated ornamentation in the pollen of *Sorbus* species [30,35,39,40]. Overall, our findings are consistent with previous palynology studies on *Sorbus*, but there are some variations at the subgenus and species levels.

Five *Sorbus* species from Poland belonging to subgenera *Sorbus*, *Aria*, *Chamaemespilus*, and *Torminaria* were reported to have pollen morphology that aligns with their systematic classification, with the exception of subgenus *Aria*, which exhibited different pollen morphology. The other four subgenera could be easily differentiated based on pollen morphology [36]. However, our study did not confirm this conclusion. There was a high degree of similarity in pollen morphology among the six subgenera. Species within subgenus *Sorbus* displayed five different ornamentations and shapes, while subgenera *Torminaria* and *Chamaemespilus* exhibited ornamentations and shapes that were distinct from subgenus *Sorbus*. Subgenus *Aria* showed no distinctive pollen characters, and it encompassed five different pollen shapes and three types of ornamentation.

### 3.3. The Comparison of Pollen Morphology, Molecular Systematics, and Morphological Classification

Molecular phylogeny and morphology have provided evidence to support the notion that *Sorbus s.l.* is a polyphyletic genus [20,21,22,23]. Previous studies have also identified five groups within *Sorbus s.l.* [2,18,42], and morphological data have been categorized into three or six subgenera [1,16]. However, when conducting cluster analysis, the taxa from different subgenera did not form the expected clades. Group B consisted of nineteen species belonging to subgenus *Sorbus*, while the remaining five groups had individuals from at least three subgenera. Therefore, the pollen morphology does not align with the taxonomic relationships inferred from molecular phylogenies and morphology analysis.

It has been previously demonstrated that *Sorbus s.s.* (subgenus *Sorbus*) is a monophyletic group, forming a distinct phylogenetic lineage with species from the temperate zone of the northern hemisphere [19,20,43,44]. Furthermore, subgenus *Sorbus* is believed to have originated from the primitive *Aria* [42,45]. There were no significant differences in pollen morphology between subgenera *Sorbus* and *Aria*. Although nineteen species from subgenus *Sorbus* formed a single branch on the cluster tree, the other sixty-one species from six subgenera were intertwined in different branches. Therefore, the claim of subgenus *Sorbus* being monophyletic cannot be supported by pollen morphology alone.

### 3.4. Interspecific Clustering of Different Subgenera Based on Pollen Morphology

*Sorbus s.l.* includes both simple-leaved and compound-leaved taxa. Some taxonomists have divided the simple-leaved taxa into two genera (*Aria* and *Micromeles*) or merged them into one genus (*Aria*). Subgenus *Aria* mainly occurs in Europe and Asia. Initially, Persoon considered *Aria* to be a subgenus of *Sorbus* [1,46]. However, later taxonomic studies placed it in *Pyrus* or *Sorbus* as a section [9]. Host treated it as a separate genus [47], and this treatment was followed by Sennikov and Kurtto, who proposed five hybridogenous genera with simple leaves [2]. The instability of the carpel number and free top of the ovary were considered unique characteristics of the *Aria* and *Aucuparia* groups [42]. Rushforth described five new genera of simple leaves based on references in the literature [25]. However, the species within the simple-leaved taxa exhibited multiple types in pollen morphology. The majority of the group (70.73%) exhibited striate sculptures, with over half of the species (51.85%) being medium-sized. Different subgenera or genera described by taxonomists do not exhibit distinct characteristics in terms of pollen.

Subgenus *Aria* consists of some simple-leaved taxa of *Sorbus s.l.* and mainly occurs in Europe. Scholars have classified the species of subgenus *Aria* into five (*Aria*, *Griffitharia* Rushforth, *Wilsonaria* Rushforth, *Micromeles* Decaisne, and *Alniaria* Rushforth) or four (*Aria* Host, *Karpatiosorbus* Sennikov & Kurtto, *Hedlundia* Sennikov & Kurtto, and *Borkhausenia* Sennikov & Kurtto) different genera [2,25]. The pollen characteristics of subgenus *Aria* observed in this study show no obvious regularity. In the cluster analysis results, *Aria* and other subgenera are often clustered together, but some species of *Aria* can form a small single branch. The results of biological research indicate that *S. torminalis* and *S. aucuparia* are likely involved in the speciation of *S. tauricola*. However, the pollen morphology of these species shows distinct differences, enabling their differentiation, and the intergroup distance between them is considerable.

Subgenus *Micromeles* is endemic to Asia and differs from *Sorbus* in terms of style and carpel structures [6,20,45]. Previously, *Micromeles* was classified under *Aria* due to its similarity in fruit structure [48]. Rushforth divided the species of subgenus *Micromeles* into four genera: *Thomsonaria* Rushforth, *Alniaria* Rushforth, *Micromeles* Decaisne, and *Dunniaria* Rushforth [25]. In this study, the examination of pollen morphology revealed a variety of shapes and ornamentations. Species of subgenus *Micromeles* formed no more than two species per branch, and species within subgenus *Micromeles* were scattered throughout the phenogram. The pollen morphology of *Sorbus* was insufficient in elucidating the taxonomic relationships among subgenus *Micromeles* species.

Subgenus *Sorbus*, also known as *Sorbus s.s.*, consists of species with compound-leaves. Both molecular phylogeny and morphology studies have confirmed its monophyly [15,19,23,43]. No distinct patterns were observed, and the majority of species demonstrated considerable variations in pollen morphology within this subgenus. Except for 19 species that formed a well-defined clade, other taxa within the subgenus were scattered in the cluster analysis tree. The significant differences in pollen characteristics between species also provide useful information for their identification. For instance, *S. koehneana* was previously considered a synonym or variant of *S. multijuga* [12]; however, they exhibit different pollen shapes as observed in this study.

Various studies have been reported on the subgenera *Torminaria*, *Chamaemespilus*, and *Cormus*. Clustering analysis has indicated that subgenus *Torminaria* has an isolated position within *Sorbus* [45]. However, our data reveal that it cannot be separated from other subgenera. In terms of morphological characters such as flowers and petals, subgenus *Chamaemespilus* differs from all other subgenera of *Sorbus* [42]. Species within subgenera *Torminaria* and *Chamaemespilus* were mistakenly placed in *Crataegus* due to the special character differences of *Sorbus*. While the vegetative parts of subgenus *Cormus* closely resemble subgenus *Sorbus*, they can be distinguished by flower and fruit characters. Some studies have reported that subgenus *Cormus* is distantly related to *Sorbus* [2,45]. Additionally, the hole density of subgenus *Cormus* species is slightly larger than that of species from subgenus *Torminaria*, making it easier to distinguish between the two subgenera. The pollen shape and ornamentation of subgenus *Chamaemespilus* show a different type compared to subgenera *Torminaria* and *Cormus*. In our cluster analysis, these three subgenera were grouped together (Group C). Previous studies have shown that *S. intermedia* contains apigenin O-glucuronide, which reflects its close affinity to *S. torminalis* [4,45]. The cluster phenogram also indicates that *S. intermedia* and *S. torminalis* share the same branch, indicating a closely related pollen morphology between the two species.

The intersubgeneric and interspecific classification of *Sorbus*, as determined by morphology and molecular phylogeny, did not align with the findings of pollen morphology. Subgenera *Torminaria*, *Chamaemespilus*, and *Sorbus* have the capacity to hybridize with *Aria*, indicating that certain *Sorbus* species derive from intersubgeneric hybridization. The phenogram analysis based on pollen statistics can serve as supporting evidence for interspecific relationships between some hybrid species and their parent species. Notably, certain sibling species that were difficult to differentiate using traditional taxonomic classification exhibited distinct pollen characteristics, such as *S. tapashana* and *S. tianschanica*, *S. folgneri*, and *S. hemsleyi*. Additionally, numerous species assigned to different subgenera or with distant genetic relationships clustered together in the same branch of the phenogram, including *S. domestica*, *S. keissleri*, *S. buschiana*, and *S. gracilis*. These results suggest that pollen morphology in *Sorbus* may evolve in diverse patterns.

## 4. Materials and Methods

### 4.1. Sample Collection

A total of eighty species from six subgenera, following the classification of Phipps et al. [1], were collected. Sixty pollen samples were obtained from fresh collections or collected from various herbaria mentioned in Supplementary Table S2. The remaining 20 species’ pollen data were sourced from Jing and Yang [35,39].

### 4.2. Pollen Morphological Characteristics

Dried pollen grains were mounted on stubs and coated with gold at 10 mA for 1 min using an ion-sputtering device. The morphological features of the pollen grains were observed using an environmental scanning electron microscope (Quanta 200, FEI company, Shanghai, China) at a 10 kV accelerating voltage at Nanjing Forestry University. For species with wide distribution, at least two samples from different areas were scanned. Twenty pollen grains were randomly selected, and their equatorial axis length (E), polar axis length (P), colpi length (C), and hole density were measured using digital SEM images processed with Image J 1.53 [49]. The ratios of colpi length to polar axis length (C/P), polar axis length to equatorial axis length (P/E), as well as the mean values of P and E, were calculated using Excel.

### 4.3. Cluster Analysis

Three qualitative and four quantitative variables were chosen for cluster analysis, which was performed using IBM SPSS Statistics 25 software. The terminology for pollen shape and ornamentation adhered to Erdtman [50], Wang et al. [51], and Halbritter et al. [52]. The hole density was coded based on the average value of actual measurements for each pollen grain. The pollen shape (P/E) was categorized into five types: suboblate, spheroidal, subprolate, prolate, and perprolate. The five types of pollen grain ornamentation included: striate-perforate, striate, cerebroid-perforate, cerebroid, and foveolate (Figure 7 and Table 2).

## 5. Conclusions

The importance of SEM studies for accurate and efficient identification of *Sorbus s.l.* using various palyno-morphological characters has been demonstrated in this study. It has been concluded that there is a high diversity pattern in the pollen of *Sorbus*. With the exception of subgenera. *Cormus*, *Torminaria*, and *Chamaemespilus*, there are no unique pollen morphologies for any of the subgenera or species, due to the presence of overlapping characters among these subgenera and taxa.

While pollen morphology alone is insufficient to fully elucidate or reconstruct the taxonomic relationships within *Sorbus* at the sub-generic or sectional level, it can provide valuable information for further taxonomic treatment at the specific level.

This study presents the first comprehensive analysis of *Sorbus* in terms of pollen morphology. However, the results do not support the monophyly of the six subgenera. For future investigations, it is recommended to increase the sample size and conduct more extensive research.

## Figures and Tables

**Table 1:** Distribution of subgenus pollen morphology.

Characters	Subgenus (Number of Species)						
	*Aira*	*Chamaemespilus*	*Cormus*	*Micromeles*	*Sorbus*	*Torminaria*	Total
Ornamentation Type I	10	0	0	2	17	0	29
Ornamentation Type II	5	0	0	2	18	0	25
Ornamentation Type III	3	1	0	4	4	0	12
Ornamentation Type IV	0	0	0	1	6	0	7
Ornamentation Type V	0	0	1	0	5	1	7
Suboblate	3	0	0	1	3	0	7
Spheroidal	3	0	0	0	10	0	13
Subprolate	1	1	0	2	7	0	11
Prolate	8	0	1	6	25	1	41
Perprolate	3	0	0	0	5	0	8
Hole density (0)	5	0	0	3	24	0	32
Hole density (0.18–2.81)	12	0	0	5	25	1	43
Hole density (3.74–5.70)	1	1	1	1	1	0	5

**Table 2:** Morphological characters used in cluster analysis.

Characters	Type of Traits	Code
Length polar axis (P)	Quantitative	μm
Length of equatorial axis (E)	Quantitative	μm
Length of colpi (C)	Quantitative	μm
The ratio of colpus length to Polar axis length (C/P)	Quantitative	ratio
Pollen shape (P/E)	Qualitative	suboblate = 1; spheroidal = 2; subprolate = 3; prolate = 4; perprolate = 5
Pollen ornamentation	Qualitative	striate-perforate = 1; striate = 2; cerebroid-perforate = 3; cerebroid = 4; foveolate = 5
Hole density	Qualitative	absent = 0; 0.18–2.81/μm^2^ = 1; 3.74–5.70/μm^2^ = 2

## Data Availability

All data and materials used in this study are included in this paper.

## Supplementary Materials

The following supporting information can be downloaded at: https://www.mdpi.com/article/10.3390/plants12183318/s1, Table S1: Summary of pollen morphological data for investigated *Sorbus* taxa title; Table S2: Voucher information of genus *Sorbus* species examined in this study.
