# Supplementary material for: Pollen Morphology in Sorbus L. (Rosaceae) and Its Taxonomic Implications
Source: Plants (Basel). 2023 Sep 20;12(18):3318. doi: 10.3390/plants12183318 (PMC10534392; doi:10.3390/plants12183318)
Supplement: Supplementary file 1 [file plants-12-03318-s001.zip › Table S1.pdf]

Table S1. Summary of pollen morphological data for investigated *Sorbus* taxa.

Taxa	Polar Axis Length (P) [µm]	Equatorial Axis Length (E) [µm]	Colpi Length (C) [µm]	P/E Ratio	C/P Ratio	Pollen Shape	Ornamentation Type
**1. Subg. *Aria***							
S. albovii	30.99±4.55	22.09±7.36	25.36±3.21	1.40±0.15	0.82	4	2
S. aria	39.09±2.97	19.07±2.05	34.71±2.93	2.05±0.15	0.89	5	1
S. buschiana	28.32±3.36	17.83±3.09	23.79±2.87	1.59±0.08	0.84	4	3
S. caucasica	20.55±2.39	24.20±2.32	16.22±1.76	0.85±0.05	0.79	1	1
S. devoniensis	50.70±3.1	27.80±3.68	43.60±2.20	1.82±0.21	0.86	4	1
S. graeca	18.81±0.6	21.46±0.75	12.92±0.58	0.88±0.05	0.69	1	3
S. hemsleyi	37.18±1.03	18.58±2.23	33.42±1.35	2.00±0.22	0.90	5	1
S. hybrida	31.98±3.59	16.57±1.98	29.09±1.64	1.93±0.20	0.91	4	1
S. intermedia	40.86±9.65	22.78±4.7	32.41±3.43	1.79±0.14	0.79	4	3
S. latifolia	46.90±3.59	25.49±1.59	39.23±3.54	1.84±0.17	0.84	4	1
S. megalocarpa	38.33±2.07	18.92±1.40	35.14±1.21	2.03±0.05	0.92	5	1
S. mougeotii	44.59±3.39	24.38±2.78	37.64±2.56	1.83±0.21	0.84	4	1
S. persica	25.46±2.3	30.55±4.2	18.19±1.13	0.83±0.06	0.71	1	2
S. tauricola	32.62±3.23	32.38±1.87	25.34±1.92	1.01±0.03	0.78	2	2
S. thibetica	27.38±1.14	24.14±1.21	19.61±1.03	1.13±0.12	0.72	2	1
S. umbellata	27.50±1.2	30.13±4.47	21.41±1.11	0.91±0.12	0.78	2	2
S. verrucosa	25.39±5.46	19.67±3.57	20.87±1.36	1.29±.0.08	0.82	3	2
S. yuana	37.66±3.45	19.17±2.73	33.97±2.48	1.96±0.23	0.90	4	1
**2. Subg. *Chamaemespilus***							
S. chamaemespilus	33.67±2.4	25.49±2.45	26.93±2.33	1.32±0.07	0.80	3	3
**3. Subg. *Cormus***							

S. domestica	37.61±2.58	20.26±1.63	31.94±1.18	1.86±0.18	0.85	4	5
**4. Subg. *Micromeles***							
S. alnifolia	41.73±2.91	22.20±1.22	35.63±1.18	1.88±0.22	0.85	4	1
S. caloneura	25.97±3.52	15.35±1.93	22.73±1.87	1.69±0.14	0.88	4	2
S. corymbifera	29.52±0.81	18.90±1.56	25.63±1.15	1.56±0.07	0.87	4	3
S. dunnii	25.39±4.11	19.00±4.01	20.95±1.37	1.34±0.11	0.83	4	3
S. folgneri	22.89±1.91	26.48±1.95	14.71±0.88	0.86±0.07	0.64	1	3
S. japonica	19.43±1.33	16.80±1.94	15.03±0.48	1.16±0.06	0.77	3	4
S. keissleri	31.81±2.32	22.76±1.77	25.71±1.25	1.40±0.07	0.81	4	3
S. thomsonii	19.75±1.79	16.86±4.13	16.68±0.94	1.17±0.07	0.84	3	2
S. tsinlingensis	30.55±1.62	18.06±0.56	25.42±1.83	1.69±0.80	0.83	4	1
**5. Subg. *Sorbus***							
S. albopilosa	18.16±1.09	11.58±0.87	15.87±1.52	1.57±0.13	0.87	4	1
S. amabilis	26.45±2.72	15.49±2.68	22.77±2.85	1.78±0.12	0.86	4	1
S. americana	42.73±2.94	20.49±1.81	38.91±1.56	2.09±0.14	0.91	5	1
S. amurensis	30.47±3.34	18.03±1.73	28.29±2.31	1.69±0.11	0.93	4	1
S. aucuparia	31.47±4.5	17.44±1.51	28.62±1.20	1.80±0.07	0.91	4	1
S. boissieri	27.4±4.94	13.88±3.83	20.08±1.04	1.97±0.09	0.73	4	1
S. californica	34.43±2.77	24.56±1.95	33.81±2.08	1.40±0.11	0.98	4	2
S. commixta	36.50±1.47	19.25±1.87	33.72±1.95	1.90±0.06	0.92	4	5
S. decora	23.85±3.05	22.61±1.56	19.99±0.68	1.05±0.06	0.84	2	2
S. discolor	20.15±1.5	13.65±2.11	15.78±0.82	1.48±0.17	0.78	4	4
S. esserteauiana	32.79±1.24	16.37±0.94	29.16±2.15	2.00±0.13	0.89	5	5
S. filipes	29.7±2.04	27.59±1.68	26.03±2.36	1.08±0.12	0.88	2	1
S. glomerulata	34.35±4.62	19.92±2.28	28.96±4.45	1.72±0.22	0.84	4	2
S. gracilis	28.88±3.51	17.95±1.36	24.71±1.72	1.61±0.18	0.86	4	3

S. harrowiana	27.08±4.51	18.03±1.93	24.24±1.66	1.50±0.11	0.90	4	3
S. helenae	19.62±3.51	17.06±4.77	16.21±0.81	1.15±0.16	0.83	3	4
S. hupehensis	29.17±2.2	14.45±1.67	25.09±1.23	2.02±0.03	0.86	5	1
S. insignis	20.54±0.86	20.78±1.75	16.98±0.68	0.99±0.03	0.83	2	4
S. kiukiangensis	23.54±0.01	22.66±2.82	23.00±0.66	1.04±0.14	0.98	2	2
S. koehneana	39.18±2.46	24.59±2.28	34.47±2.39	1.59±0.83	0.88	4	2
S. kurzii	18.15±1.9	20.74±2.24	16.61±1.44	0.88±0.05	0.91	1	3
S. matsumurana	18.61±0.91	22.17±0.92	15.53±1.06	0.84±0.05	0.83	1	2
S. microphylla	25.94±2.29	24.16±1.96	22.44±1.73	1.07±0.09	0.87	2	1
S. monbeigii	30.94±1.76	21.13±1.91	27.49±1.28	1.46±0.14	0.89	4	2
S. multijuga	34.86±2.78	17.11±1.26	30.93±3.30	2.04±0.15	0.89	5	2
S. obsoletidentata	29.57±5.37	17.67±1.82	27.96±5.42	1.67±0.13	0.82	4	2
S. oligodonta	25.23±3.45	15.49±1.85	20.39±6.39	1.63±0.03	0.81	4	2
S. pohuashanensis	32.02±3.18	17.09±2.26	29.07±2.43	1.87±0.17	0.91	4	1
S. poteriifolia	22.88±1.26	21.84±0.46	18.78±2.18	1.05±0.06	0.82	2	2
S. prattii	26.67±0.68	31.95±1.26	24.09±0.79	0.83±0.08	0.90	1	4
S. pteridophylla	26.33±1.83	13.77±1.14	21.99±1.78	1.92±0.14	0.84	4	2
S. randaiensis	35.38±3.16	17.16±1.67	30.55±4.95	2.07±0.22	0.86	5	1
S. reducta	23.8±1.91	21.89±1.67	21.27±2.97	1.09±0.10	0.89	2	2
S. rehderiana	32.06±2.43	17.07±1.42	27.91±2.49	1.88±0.18	0.87	4	1
S. rufo-ferruginea	32.81±2.39	19.08±1.72	29.77±3.23	1.72±0.13	0.91	4	1
S. rufopilosa	29.33±1.48	15.06±1.01	25.45±1.80	1.96±0.18	0.87	4	2
S. sambucifolia	27.07±2.17	22.11±2.32	23.33±1.42	1.22±0.06	0.86	3	5
S. sargentiana	16.64±0.84	14.81±2.94	13.66±0.38	1.12±0.27	0.82	2	2
S. scalaris	17.99±1.06	13.77±0.98	15.08±1.19	1.31±0.08	0.84	3	2
S. scopulina	32.43±1.84	17.18±0.82	28.19±2.36	1.89±0.03	0.87	4	1

S. setschwanensis	26.96±1.50	16.55±0.25	23.10±1.16	1.61±0.08	0.86	4	1
S. sibirica	31.43±2.51	18.16±1.33	27.65±1.93	1.73±0.12	0.88	4	5
S. sitchensis	26.25±4.84	28.46±3.95	21.43±1.76	0.92±0.04	0.82	2	5
S. tapashana	25.76±3.91	18.84±0.74	23.47±4.44	1.37±0.17	0.91	4	1
S. tianschanica	25.8±1.63	18.42±1.93	20.49±0.47	1.40±0.07	0.79	4	4
S. ursina	21.31±4.77	18.46±2.66	16.86±0.75	1.15±0.07	0.79	3	3
S. vilmorinii	28.46±3.63	21.88±2.03	23.49±1.25	1.30±.016	0.83	3	4
S. wilsoniana	18.37±1.08	15.09±0.79	15.62±1.61	1.22±0.08	0.85	3	1
S. zayuensis	31.57±1.42	26.95±1.942.82	27.15±0.95	1.17±0.02	0.86	3	2
S. foliolosa	25.06±1.43	26.09±1.21	19.03±1.18	0.96±0.27	0.76	2	2
**6. Subg. *Torminaria***							
S. torminalis	40.55±5.68	22.07±3.18	35.04±2.37	1.84±0.16	0.86	4	5
