# Supplementary material for: Pollen Morphology in Sorbus L. (Rosaceae) and Its Taxonomic Implications
Source: Plants (Basel). 2023 Sep 20;12(18):3318. doi: 10.3390/plants12183318 (PMC10534392; doi:10.3390/plants12183318)
Supplement: Supplementary file 1 [file plants-12-03318-s001.zip › Table S2.pdf]

Table S2. Voucher information of genus *Sorbus* species examined in this study.

**Species Name**	**Place of Collection**	**Collector**	**Vouchers**	**Herbarium Information**
Sorbus albovii Zinserl.	Russian Federation: Sakhalin, Northwest Sakhalin Island	T Zaikonnikova	s.n.	MO
Sorbus alnifolia (Siebold & Zucc.) C. Koch	South Korea: Jeollanam-do, Taehuksan Island	M Li	AA74-90	NF
Sorbus americana Marsh	Canada: New Brunswick, Charlotte	M Li	AA1845-66	NF
Sorbus amurensis Koehne	Russia: Khabarovskiy, Nanayskiy	Anonymous	s.n.	MO
Sorbus aria (L.) Crantz	France: Grand Est, Haut-Rhin	M Li	AA115-2005	NF
Sorbus aucuparia L.	Sweden; Gotland, Hejdeby	M Li	AA1257-84	NF
Sorbus boissieri C. K. Schneid.	Georgia: Abkhazia, Anchkho Pass	D McNeal	220	MO
Sorbus buschiana Zinserl.	Georgia: South Ossetia, Dzhara district	T Zaikonnikova	5921A	MO
Sorbus buschiana Zinserl.	United States: California, Butte	L Ahart	6323	MO
Sorbus caloneura (Stapf) Rehder	China: Hubei, Shennongjia	E H Wilson	3494	MO
Sorbus caucasigena Kom. ex Gatsch.	Georgia: Ajara, Keda	R Gagnidze et al.	809	MO
Sorbus chamaemespilus (L.) Crantz	France: Alpes Cote d'Azure, Alpes-de-Haute	Anonymous	3270	MO
Sorbus commixta Hedl.	Japan: Kagoshima, Yakushima	M Li	AA1651-8	NF
Sorbus corymbifera (Miq.) T. H. Nguyên & Yakovlev	Thiland: Nakhon Si Thammarat, Na Bon	XM Zhou	s.n.	MO
Sorbus decora (Sarg.) C. K. Schneid.	USA: Maine, Aroostoo	ML Ferrcal	2305	GH00747045
Sorbus devoniensis E. F. Warb.	Netherlands: Gelderland, Wageningen	M Li	AA42-96	NF
Sorbus discolor Maxim.	China: Henan, Luoning	M Li	H928	MO
Sorbus domestica L.	Czech Republic: Central Bohemian Region, Prague	M Li	AA385-25	NF
Sorbus dunnii Rehder	China: Fujian, Wuyishan	M Li	674	MO
Sorbus esserteauiana Koehne	China: Sichuan, Dujiangyang	M Li	AA179-92	NF
Sorbus folgneri (C. K. Schneid.) Rehder	China: Sichuan, E'mei	CH Li	101	MO
Sorbus foliolosa (Wall.) Spach	China: Xizang, Bomi	FG Shem	13203	MO
Sorbus gracilis (Siebold & Zucc.) K. Koch	Japan: Gunma, Momono-mura	O Ohba	71044	MO
Sorbus graeca (Spach) Kotschy	Georgia: Kartli, Tbilisi	CG Alm et al.	2294	MO
Sorbus harrowiana (Balf. fil. & W. W. Sm.) Rehder	China: Yunnan, Gongshan	M Li	GYD0144	MO
Sorbus helenae Koehne	China: Sichuan, E'mei	HG Xu	s.n.	CDBI
Sorbus hemsleyi (C. K. Schneid.) Rehder	China: Hubei, Shennongjia	M Li	AA1981-80	NF
Sorbus hupehensis C. K. Schneid.	China: Hubei, Shennongjia	M Li	AA1675-80	NF
Sorbus hybrida (L.) L.	Sweden: Gotland, Visby	M Li	AA430-93	NF
Sorbus insignis (Hook. fil.) Hedl.	China: Xizang, Motuo	B Xu et al.	YLZB4493	CDBI
Sorbus intermedia (Ehrh.) Pers.	Sweden: Smaland, Alghult	M Li	AA476-85	NF

Sorbus japonica (Maxim.) Koehne	Japan: Kochi, Ochi-cho	T Takahashi	317	MO
Sorbus keissleri (C. K. Schneid.) Rehder	China: Sichuan, Luding	M Li	WH76	CDBI
Sorbus koehneana C. K. Schneid.	China: Sichuan, Luding	GH Wang	91020	MO
Sorbus kurzii (Watt ex Prain) C. K. Schneid.	China: Yunnan, Tengchong	LZ Hevy	11868	MO
Sorbus latifolia (Lam.) Pers.	Spain: Vizcaya, Amorebieta	M Li	AA18462	NF
Sorbus matsumurana (Makino) Koehne	Japan: Hokkaido, Kamikawa	T Kawahara	691	MO
Sorbus monbeigii (Cardot) N. P. Balakr.	China: Yunnan, Deqin	M Li	145	CDBI
Sorbus mougeotii Godr. & Soy.-Will.	Sweden: Skane, Brantevik	M Li	AA559-48	NF
Sorbus persica Hedl.	Uzbekistan: Tashkent, Gazalkent	Anonymous	4461	MO
Sorbus pohuashanensis (Hance) Hedl.	China: Heilongjiang, Fusong	M Li	AA1175-80	NF
Sorbus prattii Koehne	China: Sichuan, Kangding	M Li	LMJXZC056	CDBI
Sorbus rehderiana Koehne	China: Sichuan, Dujiangyang	M Li	AA443-95	NF
Sorbus rufo-ferruginea C. K. Schneid.	Japan: Hondo, Lake Yumato	M Li	AA364-80	NF
Sorbus sambucifolia (Cham. & Schltdl.) M. Roem.	Japan: Yamagata, Asahi-machi	Anonymous	7003	MO
Sorbus sargentiana Koehne	China: Sichuan, Baoxing	M Li	192	CDBI
Sorbus scopulina Greene	United States: Idaho, Bonner	M Li	AA310-75	NF
Sorbus sibirica Hedl.	Russian Federation: Sakhalin, Northwest Sakhalin Island	M Li	AA422-95	NF
Sorbus sitchensis M. Roem.	USA: Washington, King County	PF Zika	22792	MO
Sorbus tauricola Zaik.	Ukraine: Crimea, Jalta	T Zaikonnikova	7011	MO
Sorbus thibetica (Cardot) Hand.-Mazz.	China: Yunnan, Lijiang	M Li	SBB1187	CDBI
Sorbus thomsonii (King ex Hook. fil.) Rehder	China: Sichuan, E'mei	EH Wilson	4867	MO
Sorbus tianschanica Rupr.	China: Xinjiang, Urumchi	ZM Zhu	s.n.	CDBI
Sorbus torminalis (L.) Crantz	France: Bourgogne-Franche-Comte, Fontaine Francaise	M Li	AA246-98	NF
Sorbus tsinlingensis C. L. Tang	China: Shaanxi, Taibai	M Li	AA544-88	NF
Sorbus umbellata (Desf.) Fritsch	Greece: Crete, Nomos Rethimnis	NJ Turland	1583	MO
Sorbus ursina (Wall.) Decne.	Nepal: Bagmati, Rasuwa	M Suzuki	8540111	KATH
Sorbus verrucosa (Decne.) Rehder	Vietnam: Cao Bang, Nguyen Binh	PH Hoang	1272	MO
Sorbus vilmorinii C. K. Schneid.	China, Xizang, Markam	M Li	MK30789	CDBI
Sorbus yuana Spongberg	China: Hubei, Shennongjia	M Li	AA1894-80	NF
